# Interventions to improve emergency department use for mental health reasons: protocol for a mixed-methods systematic review

**DOI:** 10.1186/s13643-019-1008-6

**Published:** 2019-04-03

**Authors:** Amanda Digel Vandyk, Mark Kaluzienski, Catherine Goldie, Yehudis Stokes, Amanda Ross-White, Jeremy Kronick, Matthew Gilmour, Colleen MacPhee, Ian D. Graham

**Affiliations:** 1 School of Nursing, Faculty of Health Sciences, University of Ottawa, Ottawa, Canada; 2 Department of Psychiatry and Psychiatric Emergency Services, The Ottawa Hospital, Ottawa, Canada; 3 School of Nursing, Queen’s University, Kingston, Canada; 4 Clinical Outreach Services Librarian, Queen’s University, Kingston, Canada; 5 C.D. Howe Institute, Toronto, Canada; 6 University of Ottawa, Ottawa, Canada; 7 Clinical Epidemiology Program, Ottawa Hospital Research Institute, Ottawa, Ontario Canada; 8 Community Mental Health Crisis Services, The Ottawa Hospital, Ottawa, Ontario Canada; 9 School of Epidemiology and Public Health, School of Nursing (cross-appointed), University of Ottawa, Ottawa, Canada; 10 Centre for Practice-Changing Research, The Ottawa Hospital Research Institute, Ottawa, Canada

**Keywords:** Psychiatry/mental health, Emergency department use, Interventions, Systematic review, Mixed-methods

## Abstract

**Background:**

Healthcare resources are limited and unnecessary, and inappropriate emergency department use is now a highly visible healthcare priority. Individuals visiting the emergency department for mental health-related reasons are often amongst the most frequent presenters. In response, researchers and clinicians have created interventions to streamline emergency department use and several primary studies describe the effects of these interventions. Yet, no consensus exists on the optimal approach, and information on the quality of development, effectiveness, acceptability, and economic considerations is hard to find. The purpose of this study is to systematically review interventions designed to improve appropriate use of the emergency department for mental health reasons.

**Method:**

A mixed-method systematic review using Joanna Briggs Methodology. Search combining electronic databases (EMBASE, MEDLINE, PsycINFO, CINAHL, HealthSTAR, PROQUEST, Cumulative Index to Nursing and Allied Health) and secondary searches (grey literature and hand search with consultation). Two independent reviewers will screen titles and abstracts using predetermined eligibility criteria and a third reviewer will resolve conflicts. Full texts will also be screened by two independent reviews and conflicts resolved in a consensus meeting with a third reviewer. A pilot-tested data extraction form will be used to retrieve data relevant to the study objectives. We will assess the quality and of all included studies. Data describing interventions will be summarized using logic models and reported narratively. Quality of development will be assessed using the Oxford Implementation Index. For data on intervention effectiveness, we will assess statistical heterogeneity and conduct a meta-analysis using a random effects method, if appropriate. For interventions that cannot be pooled, we will report outcomes narratively and descriptively. Qualitative data on acceptability will be synthesized using meta-aggregation and an economic evaluation of interventions will be done. The reporting of this protocol follows the PRISMA-P statement.

**Discussion:**

Using a combined systematic review methodology and integrated knowledge translation plan, the project will provide decision makers with concrete evidence to support the implementation and evaluation of interventions to improve emergency department use for mental health reasons. These interventions reflect widespread priorities in the area of mental health care.

**Systematic review registration:**

PROSPERO CRD42018087430

## Background

While emergency departments (ED) offer accessible care 24 h-a-day and act as a portal of entry for patients requiring immediate healthcare services, overcrowding and increasing yearly visit rates are a worldwide problem [1, 2]. Visits for mental health-related reasons are known to significantly contribute to overcrowding, with reports indicating that up to 20% of visits are for mental health needs [3, 4]. Although many mental health issues (such as acute psychosis or mania) require emergency care, other issues, often instigating ED visits, may be better managed in the community through primary care and auxiliary services [5]. Inappropriate and unnecessary use of the ED has negative implications economically, organizationally, and for health professionals providing care. Furthermore, inefficient use of the ED or ED care that does not meet patient needs negatively affects these persons, their health and recovery, as well as their support systems [6].

The widespread recognition of the impact of frequent ED use by people with mental health issues has led to the publication of several primary studies and one systematic review [7]. This review explored the type and effectiveness of interventions designed to reduce ED visits. The review included 11 studies (1985–2009), and the authors concluded that interventions using a case management approach reduced cost and improved outcomes, though no meta-analysis was done. Unfortunately, this review does not provide adequate information to inform ED practice for patients with mental illness, because population-specific interventions—including those designed specifically for persons with mental health needs—were excluded. Furthermore, only quantitative studies were included and thus patient and provider perspectives of the interventions were not examined. In preparation for this protocol, we attempted to replicate and update the Althaus search to ascertain how many mental health-related interventions were either missed or published after the search date. In screening the initial 100 hits, we identified 12 studies describing such interventions (e.g., [8]). Clearly, a significant body of evidence exists that contains detailed information about the needs of persons who visit the ED for mental health-related reasons.

In the current healthcare context where financial and human resources are limited, the burden of unnecessary and inappropriate emergency department (ED) use is apparent. This has implications at all levels including patients, healthcare professionals, and policymakers. In response, researchers and clinicians have attempted to develop interventions to streamline ED use. Several primary studies describe the effects of these interventions, and yet no consensus exists on the optimal approach, including quality of development, effectiveness, acceptability, and economic considerations. The *aim* of this study is to conduct a systematic review of interventions designed to improve appropriate use of the ED for mental health (including substance use)-related reasons and to assess the relevance and feasibility of implementing these interventions.

Specifically, the study *objectives* are:

1. To describe interventions designed to improve the appropriate use of the ED for mental health-related reasons
2. To report on the quality of development, effectiveness, acceptability, and economic considerations of the identified interventions

## Methods

### Design

Our approach to this study will be a *systematic review* using the Joanna Briggs Methodology for Mixed Methods Systematic Reviews [9]. The review protocol was developed according to the PRISMA-P Statement [10] and registered with PROSPERO (CRD42018087430).

### Eligibility criteria

Eligibility criteria were created using PICO (Population, Intervention, Comparison, Outcome), as specified in the Joanna Briggs Institute (JBI) methodology, and include the following:

*Population*: Adults (persons aged 17 years or older) who visit the ED for mental health-related reasons, *excluding* child, adolescent, or cognitive impairment-only participants because healthcare and services for these populations are delivered differently.

*Intervention*: All forms of interventions about the use of the ED for mental health reasons and/or any intervention in which ED use related to a mental health reason is a measured outcome. An intervention is defined as “an act performed for, with or on behalf of a person or population whose purpose is to assess, improve, maintain, promote or modify health, functioning or health conditions” (World Health Organization, 2019). For example, intensive case management and telephone follow-up post-discharge, *excluding* the following: any intervention with a different purpose or articles reporting of system-level reform, such as deinstitutionalization or Medicare.

*Comparison*: Other treatment intervention or normal care.

*Outcome*: Quality of development, effectiveness, acceptability, and economic considerations for interventions designed to improve ED use for mental health-related reason.

*Types of studies*: To determine the *effectiveness* (or report on this outcome narratively if required) of the interventions, we will search for the following: randomized controlled trials, quasi-experimental studies, cohort studies, case-control studies, case series, and cross-sectional studies. To better understand how people perceive and experience the interventions (i.e., *acceptability*), we will search for the following: process evaluations and qualitative studies. To evaluate the *economic considerations* of the interventions, we will search for the following: reports on economic evaluations of all types, *excluding* the following: all non-research literature (e.g., expert opinion, discussion articles).

*Language*: English, French, and any other language able to be translated into English or French.

*Country*: No limits.

### Search and selection of articles

Our search strategy combines systematic searches of multiple electronic databases with secondary searches:

1. Formal search of online databases: MEDLINE, CINAHL, PsycINFO, EMBASE, Nursing and Allied Health, HealthSTAR, and PROQUEST
2. Secondary searches: grey literature search, hand search, and consultation (described below)

#### Online database search strategy

In consultation with our library scientist, we designed and executed a pilot search in the OVID MEDLINE database. This strategy included keywords and MESH headings based on our eligibility criteria and was used to gauge the likely number of studies eligible for inclusion. Twelve hundred and thirty-six MEDLINE citations were retrieved, of which approximately 135 passed first-level screening. From this, the library scientist finalized the search strategy and translated into all databases (Table 1).

**Table 1:** Search strategy

Database: Ovid MEDLINE(R), Ovid MEDLINE(R) Daily and Epub Ahead of Print, in-process and other non-indexed citations <1946 to present>	
1	exp Health Services Misuse/ (9755)
2	“Utilization Review”/ (8267)
3	((frequent or high or heavy or repeat*) adj4 (use* or flyer* or attend* or utiliz* or utilis*)).mp. (102179)
4	(repeater* or recidivis* or “revolving door*” or misus* or hyperus*).mp. (25820)
5	Emergencies/ (38832)
6	Emergency Medical Services/ (38687)
7	exp Emergency Service, Hospital/ (65719)
8	Emergency Medicine/ (12074)
9	Emergency Nursing/ (6676)
10	Evidence-Based Emergency Medicine/ (367)
11	((emergenc* or urgen* or accident*) adj (service* or department* or unit* or ward* or room*)).mp. (118324)
12	or/1-4 (139979)
13	or/5-11 (200610)
14	12 and 13 (4919)
15	mental disorders/ or exp anxiety disorders/ or exp “bipolar and related disorders”/ or exp “disruptive, impulse control, and conduct disorders”/ or exp dissociative disorders/ or exp elimination disorders/ or exp “feeding and eating disorders”/ or exp mood disorders/ or motor disorders/ or neurocognitive disorders/ or exp amnesia/ or exp cognition disorders/ or consciousness disorders/ or exp neurodevelopmental disorders/ or neurotic disorders/ or exp paraphilic disorders/ or exp personality disorders/ or exp “schizophrenia spectrum and other psychotic disorders”/or sexual dysfunctions, psychological/ or dyspareunia/ or gender dysphoria/ or “sexual and gender disorders”/or vaginismus/ or exp sleep wake disorders/ or exp somatoform disorders/or exp substance-related disorders/ or exp “trauma and stressor related disorders”/(1066885)
16	exp mental health services/ (91064)
17	Mentally Ill Persons/ (5991)
18	((anxiety or behav* or depress* or stress* or attention* or substance or alcohol*) adj3 disorder*).mp. (353126)
19	15 or 16 or 17 or 18 (1170078)
20	14 and 19 (1066)
Database: PsycINFO <1806 to October Week 1 2017>	
1	medical overuse.mp. (2)
2	unnecessary procedure.mp. (2)
3	health services misuse.mp. (1)
4	exp Utilization Reviews/ (120)
5	((frequent or high or heavy or repeat*) adj4 (use* or flyer* or attend* or utiliz* or utilis*)).mp. (26448)
6	(repeater* or recidivis* or “revolving door*” or misus* or hyperus*).mp. (20699)
7	1 or 2 or 3 or 4 or 5 or 6 (46749)
8	exp emergency services/ (7184)
9	((emergenc* or urgen* or accident*) adj (service* or department* or unit* or ward* or room*)).mp. (14443)
10	8 or 9 (14443)
11	7 and 10 (691)
12	mental disorders/ or adjustment disorders/ or exp affective disorders/ or alexithymia/ or exp anxiety disorders/ or autism spectrum disorders/or exp chronic mental illness/ or exp dissociative disorders/or exp eating disorders/ or elective mutism/ or exp factitious disorders/ or exp gender identity disorder/ or exp hoarding disorder/ or exp hysteria/ or impulse control disorders/ or koro/ or mental disorders due to general medical conditions/ or exp neurosis/ or exp paraphilias/ or exp personality disorders/ or exp psychosis/ or schizoaffective disorder/ (478707)
13	exp Mental Health Services/ (38546)
14	mentally ill offenders/ (3464)
15	((anxiety or behav* or depress* or stress* or attention* or substance or alcohol*) adj3 disorder*).mp. (180401)
16	12 or 13 or 14 or 15 (582588)
17	11 and 16 (247)
Database: Embase Classic+Embase <1947 to 2017 October 03>	
1	medical overuse.mp. (51)
2	unnecessary procedure/ (2617)
3	health services misuse.mp. (35)
4	“utilization review”/ (64764)
5	((frequent* or high or heavy or repeat*) adj4 (use* or flyer* or attend* or utiliz* or utilis*)).mp. [mp=title, abstract, heading word, drug trade name, original title, device manufacturer, drug manufacturer, device trade name, keyword, floating subheading word] (190373)
6	(repeater* or recidivis* or “revolving door*” or misus* or hyperus*).mp. (31682)
7	1 or 2 or 3 or 4 or 5 or 6 (286188)
8	exp emergency/ (54324)
9	exp emergency health service/ (85981)
10	hospital emergency service/ (1474)
11	emergency ward/ (104505)
12	emergency medicine/ (36187)
13	emergency nursing/ (6004)
14	((emergenc* or urgen* or accident*) adj (service* or department* or unit* or ward* or room*)).mp. (162592)
15	8 or 9 or 10 or 11 or 12 or 13 or 14 (295757)
16	7 and 15 (11349)
17	mental disease/ or exp addiction/ or adjustment disorder/ or alexithymia/ or exp anxiety disorder/ or exp autism/ or exp behavior disorder/ or exp dissociative disorder/ or emotional disorder/ or mental instability/ or exp mood disorder/ or exp neurosis/ or organic brain syndrome/ or organic psychosyndrome/ or exp personality disorder/ or exp psychosexual disorder/ or exp psychosis/ or exp psychosomatic disorder/ or psychotrauma/ or stupor/ or exp thought disorder/ (1606451)
18	mental health service/ (52382)
19	mental patient/ (26359)
20	((anxiety or behav* or depress* or stress* or attention* or substance or alcohol*) adj3 disorder*).mp. (310862)
21	17 or 18 or 19 or 20 (1668884)
22	16 and 21 (2071)
23	limit 22 to child <unspecified age> (210)
24	limit 22 to (adult <18 to 64 years> or aged <65+ years>) (1107)
25	23 and 24 (112)
26	22 not 23 (1861)
27	25 or 26 (1973)
28	limit 27 to (letter or note or patent) (59)
29	27 not 28 (1914)

Literature will be searched from 1990 onwards. During the 1990s, in the aftermath of the deinstitutionalization movement, rates of ED use for mental health-related reasons skyrocketed with reports indicating nearly a 40% increase in visits for mental health reasons compared to an 8% increase in overall ED use [11]. No other limits were placed on the searches. The completed search was executed on October 3, 2017, in all databases. A secondary search designed to identify newly published articles will occur just prior to completion of the review. Our library scientist will lead this step.

#### Grey literature search strategy

We will work closely with our library scientist to design a rigorous grey literature search strategy. This will use a subject-based approach [12] and consider all possible sources of information, such as organizational websites, blogs, conference proceedings, annual reports, and others. A preliminary scan of the online grey literature and discussion with our KUs revealed a substantial body of grey literature eligible for inclusion. For example, the CADTH report on *Prioritization of Care in the Emergency Department using Alternate Triage Strategies: Effectiveness and Guidelines* [13] is one such publication.

#### Hand search and consultation process

As a final step in our search strategy, we will conduct a hand search of references lists for all included studies and relevant (but not included) reviews, reference lists of studies citing the included studies, content from selected journals, and consult regularly with KUs to ensure that all eligible and pertinent information is considered.

#### Audit Trail

We have a detailed record of all search strategy procedures to ensure a transparent and replicable process. This includes the following: databases searched, subject headings, and keywords used for each database. We will maintain a similar audit trail for the grey literature and hand search sources. We will report the screening and selection process using the PRISMA flow chart.

#### Selection process

Two reviewers will independently engage in the study selection process to reduce the possibility of rejecting relevant reports [10]. We will use Covidence©, an online citation screening tool, to facilitate and monitor study selection. This software program allows for complete screening of citations, clearly displays differences in reviewers’ ratings and will help mitigate any issues pertaining to inter-rater reliability.

After removing duplicate citations, we will select studies for inclusion based on a two-level screening process. First, two reviewers will independently screen titles and abstracts for congruence with eligibility criteria (first-level screening). At this stage, we will compare results using Covidence© data and calculate a kappa statistic to assess inter-rater reliability. A meeting will be held to discuss all instances of discrepancy, and all potentially relevant citations and those with insufficient information to determine eligibility will be retained. Second, two reviewers will independently screen the full texts of the retained articles for congruence with eligibility criteria (second-level screening). Using Covidence©, the two reviewers will document the reasons for excluding citations. A final consensus meeting will be held to re-assess inter-rater reliability and to agree upon the final set of eligible citations.

### Quality appraisal of included studies

.Two reviewers will independently assess the quality of each included study using the Joanna Briggs Critical Appraisal Tools [14]. For quantitative studies, these will include the Checklist for Randomized Controlled Trials, Checklist for Quasi-Experimental Studies, Checklist for Cohort Studies, Checklist for Case Control Studies, Checklist for Case Series, and the Checklist for Analytical Cross Sectional Studies. For qualitative studies, we will use the Checklist for Qualitative Research, and for reports of economic evaluations, we will use the Checklist for Economic Evaluations. We will report on the complete critical appraisal results to provide recommendations to advance research in this area and *conduct a sensitivity analysis* to evaluate the effect of including and excluding low-quality studies on the results, if appropriate. The project lead will oversee this process and consult, when necessary, to settle discrepancies in ratings.

### Data extraction process

We will develop a fillable form in Microsoft Word for data extraction, and pilot test our template for inter-rater reliability using the first three included articles. Once finalized, two reviewers will independently extract data from each included article using this template. The data extracted will include information on the study characteristics, participant characteristics, intervention characteristics using the Template for Intervention Description and Replication (TIDieR) Checklist [15], quality of development indicators, information on cost and other economic considerations, and outcomes (effect on ED use and other author-identified outcomes). When sufficient information is not clearly reported in the published studies, we will search for secondary publications and/or contact the authors for details.

### Data synthesis

To address *objective 1* (describe interventions designed to improve appropriate use of the ED for mental health reasons), we will aggregate multiple reports on individual interventions so that each intervention (rather than each study) is a unit of interest in the review.

Using synthesis tables and narrative summaries, we will report on the intervention characteristics, benefits, challenges, and implications for use. We will also draft a logic model [16] for each intervention based on this information. A logic model is a graphic representation (a map) that facilitates the understanding of how an intervention works, by illustrating the relationships between components and the mechanisms of action (i.e., resources/inputs, activities, outputs, outcomes, impact). Logic models facilitate the transparency of systematic review findings and enhance their interpretability for decision makers [17].

To address *objective 2*, we will report on the quality of development, effectiveness, acceptability, and economic considerations of each intervention. We will again aggregate multiple reports on individual interventions so that each intervention (rather than each study) is a unit of interest in the review.

#### Quality of development

Currently, there is no accepted process to assess the quality of complex interventions. For this study, we propose the use of the Oxford Implementation Index (OII) [18]. The OII is a tool designed to capture information in four domains: (1) intervention design, (2) actual delivery by trial practitioners, (3) uptake of the intervention by participants, and (4) contextual factors. We have obtained permission for its use in this study.

#### Effectiveness

If we obtain a series of interventions and outcomes amenable to meta-analysis [19], we will assess statistical heterogeneity and pool the results using a random effects method. We will report on the relative risk (with 95% confidence interval), absolute risk reduction, and the number needed to treat for all types of interventions amenable to meta-analysis. For interventions that cannot be pooled, we will report quantitative outcomes narratively and descriptively.

#### Acceptability

We will use data from qualitative studies to assess the acceptability of the interventions (i.e., the perceptions and experiences of patients and practitioners regarding the intervention). This information will be analyzed according to the JBI meta-aggregation approach and be reported narratively and descriptively.

#### Economic evaluation

We will conduct an economic evaluation for all interventions when information is available in published articles or through contacting primary authors. Based on our knowledge of this area, it is likely that we will obtain incomplete economic data for most interventions. As such, we will use a narrative and descriptive approach to synthesize information, led by Dr. Kronick (Economics). Further, we will provide recommendations for improving the reporting of this information and discuss the potential costs of the identified interventions with our knowledge users.

## Discussion

Decreasing inappropriate and unnecessary ED use is now a highly visible healthcare priority, with nationwide attention in the area of mental health care. This review will provide decision makers with concrete evidence to support the implementation and evaluation of interventions to improve emergency department use for mental health reasons. Outputs will include a catalog of effective interventions, information on the quality of development, economic considerations, and their relevance and feasibility of implementation.

### Potential limitations

Two possible limitations, which we are likely to encounter, include (1) heterogeneity of interventions eligible for inclusion and incomplete information reported about the interventions in the literature. Recognizing these possibilities, which will limit our ability to statistically compare the effectiveness of interventions, we designed our data analysis strategy to allow for narrative summary of available information and plan to request additional information, if available, from primary authors. This approach was determined in collaboration with our knowledge users. Should we encounter a situation where we need to deviate from our protocol, we will document and report the change in the completed review [20].

## Abbreviations

ED: Emergency department
iKT: Integrated knowledge translation
JBI: Joanna Briggs Institute
KU: Knowledge users
OII: Oxford Implementation Index
PRISMA-P: Preferred Reporting Items for Systematic Review and Meta-Analysis Protocols

## References

[1] Elder E, Johnston ANB, Crilly J (2015). Review article: systematic review of three key strategies designed to improve patient flow through the emergency department. Emerg Med Australas.

[2] Pines JM, Hilton JA, Weber EJ, Alkemade AJ, Al Shabanah H, Anderson PD (2011). International perspectives on emergency department crowding. Acad Emerg Med.

[3] Li G, Lau JT, McCarthy ML, Schull MJ, Vermeulen M, Kelen GD (2007). Emergency department utilization in the United States and Ontario, Canada. Acad Emerg Med.

[4] Pasic J, Russo J, Roy-Byrne P (2005). High utilizers of psychiatric emergency services. Psychiatr Serv.

[5] van den Berg MJ, van Loenen T, Westert GP (2016). Accessible and continuous primary care may help reduce rates of emergency department use. An international survey in 34 countries. Fam Pract.

[6] Harris B, Beurmann R, Fagien S, Shattell MM (2016). Patients’ experiences of psychiatric care in emergency departments: a secondary analysis. Int Emerg Nurs.

[7] Althaus F, Paroz S, Hugli O, Ghali WA, Daeppen J-B, Peytremann-Bridevaux I (2011). Effectiveness of interventions targeting frequent users of emergency departments: a systematic review. Ann Emerg Med.

[8] Abello A, Brieger B, Dear K, King B, Ziebell C, Ahmed A (2012). Care plan program reduces the number of visits for challenging psychiatric patients in the ED. Am J Emerg Med.

[9] The Joanna Briggs Institute (2014). Methodology for mixed methods systematic reviews. The Joanna Briggs Institute reviewers’ manual 2014.

[10] Moher D, Shamseer L, Clarke M, Ghersi D, Liberati A, Petticrew M (2015). Preferred reporting items for systematic review and meta-analysis protocols (PRISMA-P) 2015 statement. Syst Rev.

[11] Stone A, Rogers D, Kruckenberg S, Lieser A (2012). Impact of the mental health care delivery system on California emergency departments. Western J Emerg Med.

[12] Giustini, D. Finding the hard to finds: searching for grey literature – 2012 update. 2012. http://guides.library.ubc.ca/c.php?g=307422&p=2632228.

[13] Canadian Agency for Drugs and Technologies in Health (2010). Prioritization of care in the emergency department using alternative triage strategies: effectiveness and guidelines.

[14] The Joanna Briggs Institute. Critical Appraisal Tools. 2016. http://joannabriggs.org/research/critical-appraisal-tools.html. Accessed 2 Apr 2018.

[15] Hoffmann TC, Glasziou PP, Boutron I (2014). Better reporting of interventions: template for intervention description and replication (TIDieR) checklist and guide. BMJ.

[16] W.K. Kellogg Foundation. W.K. Kellogg Foundation: Logic Model Development Guide. 2004. https://www.wkkf.org/resource-directory/resource/2006/02/wk-kellogg-foundation-logic-model-development-guide. Accessed 2 Apr 2018.

[17] Anderson LM, Petticrew M, Rehfuess E, Armstrong R, Ueffing E, Baker P, et al. Using logic models to capture complexity in systematic reviews: logic models in systematic reviews. Res Synth Methods. 2011;2:33–42. 10.1002/jrsm.32 26061598

[18] Montgomery P, Underhill K, Gardner F, Operario D, Mayo-Wilson E. The Oxford Implementation Index: a new tool for incorporating implementation data into systematic reviews and meta-analyses. J Clin Epidemiol. 2013;66:874–82. 10.1016/j.jclinepi.2013.03.006 PMC3746185 23810026

[19] Higgins, JPT, Green, S. Cochrane handbook for systematic reviews of interventions*.* 2011. http://handbook.cochrane.org. Accessed 2 Apr 2018.

[20] JPT H, Green S, editors. Cochrane Handbook for Systematic Reviews of Interventions Version 5.1.0 [updated March 2011]: The Cochrane Collaboration; 2011. Available from www.handbook.cochrane.org.

